# Assessment of 4 Doses of SARS-CoV-2 Messenger RNA–Based Vaccine in Recipients of a Solid Organ Transplant

**DOI:** 10.1001/jamanetworkopen.2021.36030

**Published:** 2021-11-24

**Authors:** Nassim Kamar, Florence Abravanel, Olivier Marion, Raphaelle Romieu-Mourez, Chloé Couat, Arnaud Del Bello, Jacques Izopet

**Affiliations:** 1 Department of Nephrology and Organ Transplantation, Toulouse University Hospital, Toulouse, France; 2 Université Paul Sabatier, Toulouse, France; 3 Toulouse Institute for Inflammatory and Infectious Diseases (Infinity), Inserm, French National Centre for Scientific Research, Toulouse, France; 4 Department of Virology, Toulouse University Hospital, Toulouse, France

## Abstract

This case series study assesses whether a fourth dose of a SARS-CoV-2 messenger RNA (mRNA)–based vaccine is associated with improved anti–SARS-CoV-2 antibody response in solid organ transplant recipients in France.

## Introduction

Anti–SARS-CoV-2 antibodies have been detected in up to approximately 70% of solid organ transplant recipients who were given 3 doses of the SARS-CoV-2 vaccine.^1,2^ In France, it has been allowed to offer a fourth dose on a case-by-case basis.^3^ We assessed whether a fourth dose of the SARS-CoV-2 vaccine is associated with improved anti–SARS-CoV-2 antibody concentrations in solid organ transplant recipients in France.

## Methods

This case series study was conducted from July 1, 2021, to August 5, 2021. A fourth dose of the messenger RNA-based BNT162b2 vaccine (Pfizer-BioNTech) was given to the 37 solid organ transplant recipients, including 5 (13.5%) who had a weak response to the previous 3 doses (antibody concentration <14 binding antibody units [BAU]/mL)^4^ and 31 (83.8%) who had no response to the 3 previous doses. All participants provided oral informed consent and received approval of the medical staff (Table). According to French law (Loi Jardé), anonymous retrospective studies do not require institutional review board approval. This study followed the reporting guideline for case series.

**Table.** Clinical and Biological Characteristics of Solid Organ Transplant Recipients According to Humoral Response 1 Month After 3 Doses of mRNA-Based Vaccine

Characteristic	All patients (N = 37)	Patients seronegative before dose 4^a^		*P* value
		Remained seronegative (n = 19)	Became seropositive (n = 13)	
Gender, No. (%)				
Male	20 (54.0)	12 (63.2)	6 (46.2)	.26
Female	17 (46.0)	5 (26.3)	7 (53.8)	
Age, mean (SEM), y	60 (14)	58 (16)	60 (14)	.76
Type of organ transplant, No. (%)				
Kidney	25 (67.6)	11 (57.9)	13 (100)	.03
Heart	5 (13.5)	4 (21.1)	0	
Liver	4 (10.8)	4 (21.1)	0	
Pancreas	3 (8.1)	0	0	
Rejection in the year before vaccination, No.	0	0	0	NA
Time between vaccine and transplant, mean (SD), mo	109 (84)	79 (66)	161 (97)	.007
Induction therapy, No. (%)				
No	12 (32.4)	9 (47.4)	2 (15.4)	.12
Yes	25 (67.6)	10 (52.6)	11 (84.6)	.12
Anti–IL-2 receptor blockers	15 (60.0)	6 (60.0)	6 (54.5)	.99
Polyclonal antibodies	10 (40.0)	4 (40.0)	5 (45.5)	.99
Type of immunosuppressive regimen, No. (%)				
Calcineurin-inhibitors	32 (86.5)	17 (89.5)	10 (76.9)	.37
Tacrolimus	30 (93.8)	17 (100)	8 (80.0)	.13
Ciclosporin A	2 (6.3)	0 (0)	2 (20.0)	.13
Mycophenolic acid	32 (86.5)	15 (78.9)	12 (92.3)	.62
mTOR inhibitors	9 (24.3)	4 (21.1)	4 (30.8)	.68
Steroids	34 (91.9)	17 (89.5)	12 (92.3)	.99
Count before vaccination, mean (SD), per mm^3^				
Neutrophils	6413 (1892)	6763 (2255)	5714 (1402)	.14
Lymphocytes	1298 (742)	1126 (527)	1609 (906)	.07
CD4^+^ T cells	370 (211)^b^	351 (197)^c^	374 (144)^d^	.45
CD8^+^ T cells	393 (261)^b^	452 (271)^c^	342 (201)^d^	.23
CD19^+^ T cells	61 (59)^b^	74 (71)^c^	59 (44)^d^	.88
NK cells	202 (115)^b^	228 (133)^c^	126 (52)^d^	.11
eGFR before vaccination, mean (SD), mL/min/1.73 m^2^	45 (21)	42 (20)	45 (22)	.71
Neutrophil count before dose 4, mean (SD), per mm^3^	5357 (2070)	5647 (2227)	6046 (1438)	.57
Lymphocyte count before dose 4, mean (SD), per mm^3^	1193 (711)	947 (427)	1431 (866)	.04
eGFR before dose 4, mean (SD), mL/min/1.73 m^2^	45 (20)	43 (19)	45 (23)	.81

Abbreviations: eGFR, estimated glomerular filtration rate (Chronic Kidney Disease Epidemiology Collaboration equation); IL-2, interleukin-2; mTOR, mammalian target of rapamycin; NA, not applicable; NK, natural killer.

^a^ Comparison between patients who were seronegative before the fourth dose and who either remained seronegative or became seropositive after the fourth dose.

^b^ Data are for 26 patients.

^c^ Data are for 14 patients.

^d^ Data are for 7 patients.

The first 2 doses were given 1 month apart, the third dose was administered a mean (SD) of 57 (17) days after the second dose, and the last dose was given a mean (SD) of 65 (9) days after the third dose. Anti–SARS-CoV-2 spike protein total antibody concentrations were assessed using the Wantaï enzyme-linked immunosorbent assay test. Neutralizing antibody (NAb) titers were assessed using a live virus neutralization assay. Enzyme-linked immunospot assay measuring interferon (IFN)–γ produced by specific SARS-CoV-2 T cells was performed for 14 patients (eMethods in the Supplement). Proportions were compared using the Fisher exact test. Quantitative variables were compared by the Student *t* test or the Mann-Whitney test. A 2-sided *P* value <.05 was considered to be statistically significant. All statistical analyses were performed with Prism Software v8.1 (GraphPad).

## Results

Of 37 patients included in this case series study, 20 (54.0%) were male, with a mean (SEM) age of 60 (14) years. Anti–SARS-CoV-2 antibodies were detected in 5 of 37 patients (13.5%) before dose 4 and in 18 of 37 patients (48.6%) 1 month later (*P* = .002). Among the 5 patients who were seropositive before dose 4, the median antibody concentration increased from 4 BAU/mL (range, 1-9 BAU/mL) to 402 BAU/mL (range, 87-508 BAU/mL) at 4 weeks after dose 4 (*P* < .001) (Figure, A). Neutralizing antibody titers increased from a median of 8 IU/mL (range, 2-32 IU/mL) to 16 IU/mL (range, 4-32 IU/mL) (*P* = .07).

**Figure.**
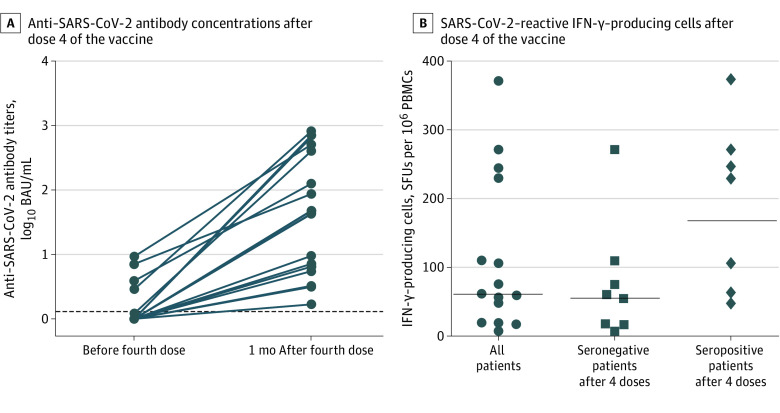
Anti–SARS-CoV-2 Antibody Concentrations and Number of SARS-CoV-2–Reactive IFN-γ–Producing Cells After a Fourth Dose of the SARS-CoV-2 Messenger RNA (mRNA) BNT162b2 Vaccine A, The dotted line represents the cutoff value. B, The numbers of cells reactive to overlapping peptide pools spanning SARS-CoV-2 structural protein S (pools S1 and S2) are shown. The numbers of SARS-CoV-2–reactive IFN-γ–producing cells in the 3 patients who had a low antibody concentration before the fourth dose were 17.5, 47.5, and 110 spot-forming units (SFUs) per 10^6^ peripheral blood mononuclear cells (PBMCs). BAU indicates binding antibody unit.

Among the 31 patients who were seronegative before dose 4, 13 (41.9%) became seropositive (median antibody concentration, 9.5 BAU/mL [range, 1.7-658 BAU/mL]) at 4 weeks after dose 4; 6 patients (19.4%) had antibody concentrations greater than 14 BAU/mL, and 2 (6.5%) had antibody concentrations greater than 140 BAU/mL (Figure, A). Among these patients, the median NAb titer was 8 IU/mL (range, 2-32 IU/mL).

At 4 weeks after dose 4, antibody concentrations were significantly higher among patients who had detectable antibodies before dose 4 than among those who had no response. However, Nab titers at 4 weeks after dose 4 did not differ between responders and nonresponders to 3 doses.

Overall, at 4 weeks after dose 4, 32 of 37 patients (86.5%) had antibody concentrations less than 140 BAU/mL (a threshold providing 12.4% protection among health care workers^4^) and all 37 patients (100%) had NAb titers less than 64 IU/mL. No breakthrough infection was observed during follow-up.

At 4 weeks after D4, the number of SARS-CoV-2–reactive IFN-γ–producing cells was 61.25 spot-forming units (SFUs) per 10^6^ peripheral blood mononuclear cells (PBMCs) (range, 2.5-372.5 SFUs per 10^6^ PBMCs), with 167.5 SFUs per 10^6^ PBMCs (range, 47.5-372.5 SFUs per 10^6^ PBMCs) among seropositive patients and 55 SFU per 10^6^ PBMC (range, 2.5-110 SFUs per 10^6^ PBMC) among seronegative patients (Figure, B).

No serious adverse event or acute rejection was observed after dose 4. One kidney transplant recipient presented with a recurrence of IgA nephropathy. Four patients presented with fatigue and myalgia. One patient indicated gastrointestinal symptoms.

## Discussion

In this case series study, our findings were similar to those of the study by Alejo et al,^5^ in which a fourth dose of SARS-CoV-2 vaccine was associated with slightly improved humoral response among patients with a weak response after 3 doses and with no improvement among those with no response after 3 doses. Neutralizing antibody titers and cellular response were low in both groups. In a study^6^ of 22 healthy volunteers (median age, 59 years), among all individuals, antibody concentrations at 1 month after 2 doses of BNT162b2 vaccine were greater than 140 BAU/mL (median, 1309 BAU/mL [range, 457-7605 BAU/mL]) and NAb titers were greater than 64 IU/mL (median 128 [range, 64-512 IU/mL]). In another study of 20 healthy volunteers (median age, 55 years), the number of SARS-CoV-2–reactive IFN-γ–producing cells at 1 month after 2 doses of BNT162b2 vaccine was 542 SFUs per 10^6^ PBMCs (range, 0-1669 SFUs per 10^6^ PBMCs) (Jacques Izopet, Pharm D, PhD, unpublished data). A limitation of our study was the small number of patients. Other strategies should be tested for solid organ transplant recipients.

## Notes

Supplement.
eMethods.
