# Supplementary material for: Assessment of 4 Doses of SARS-CoV-2 Messenger RNA–Based Vaccine in Recipients of a Solid Organ Transplant
Source: JAMA Netw Open. 2021 Nov 24;4(11):e2136030. doi: 10.1001/jamanetworkopen.2021.36030 (PMC8613594; doi:10.1001/jamanetworkopen.2021.36030)
Supplement: Supplement. — eMethods. [file jamanetwopen-e2136030-s001.pdf]

Supplemental Online Content

Kamar N, Abravanel F, Marion O, et al. Assessment of 4 doses of SARS-CoV-2 messenger RNA–based vaccine in recipients of a solid organ transplant. *JAMA Netw Open*. 2021;4(11):e2136030. doi:10.1001/jamanetworkopen.2021.36030

eMethods.

This supplemental material has been provided by the authors to give readers additional information about their work.

Serology assay

Anti-SARS-CoV-2 spike protein total antibodies were assessed before each jab using the Wantai semiquantitative microplate ELISA (Wantai SARS-CoV-2 Ab ELISA, Beijing Wantai Biological Pharmacy Enterprise CO., Ltd, Beijing, China). A positive result was defined by a signal-to-cut off (S/CO) ratio greater or equal 1.1. A linear relationship was obtained between the S/CO ratio and SARS-CoV-2 antibody concentration using the first WHO international standard (NIBSC code: 20/136, National Institute for Biological Standards, and Control, Potter Bar, Hertfordshire, EN6QG, UK). Dilutions using PBS plus 7.5% bovine serum albumin as a diluent was set up to analyse samples giving a saturated signal. Results are expressed in binding antibody units (BAU)/mL. Using this assay, we have previously reported a 100% specificity and 100% sensitivity in immunocompetent patients tested at 2 to 14 days post symptom-onset and at 15 to 45 days post symptom-onset, suggesting it as the ability to detect low level of antibodies [1].

Neutralization assay.

Neutralizing antibody titers were assessed using a live virus neutralization assay and a clinical SARS-CoV-2 strain (GISAID: EPI_ISL_804378, GISAID Clade:GH, Pango lineage: B.1.160, Nextclade: 20A.EU2) infecting Vero cells (ATCC, CCL-81™). Briefly, 10^4 cells were mixed with the virus suspension (100 TCID50) and the tested serum and incubated for 4 days in the wells of 96-well plates. Two-fold serial dilutions of each serum were tested. The plates were then examined to identify the wells showing a cytopathic effect (CPE). The titer was defined as the reciprocal of the highest serum dilution protecting cells from a CPE.

EliSpot assay

To analyze T-cell responses, enzyme-linked immunospot assay (EliSpot) measuring interferon-γ produced by specific SARS-CoV-2 T-cells were performed, the day of each dose and 1 month after the third dose. PBMCs were thawed and left to recover overnight at 37 °C (2×10^6 cells/mL) in culture medium (RPMI supplemented with glutamine, pyruvate, penicillin, streptomycin and 5% fetal calf serum). ELISpot assays were performed using plates, capture antibodies and detection reagents from the Diaclone kit for detecting IFN-γ. Cell viability was assessed by trypan blue exclusion and 0.4×10^6 viable cells were cultured for 36 h in duplicate wells with antigens in a final volume of 60 µL. The anti-HEV response was assessed with using individual 15-mers 11-aa overlapping peptide pools derived from a peptide scan through SARS-CoV-2 Spike glycoprotein (2 pools representing the S1 and S2 domains of the spike protein) (JPT-Peptide-Technologies). Results were expressed as spot forming unit (SFU)/10^6 cells. Negative control wells lacked peptides, and positive control wells included CD3/CD28 and CEF pool stimulation. The final peptide concentration was 0.25 µg/mL. Cell function was assessed by polyclonal stimulation in control wells containing 25×10^3 cells stimulated with a mix of anti-CD3 and anti-CD28 antibodies (clones HIT3a and 28.2 respectively, BD Biosciences, 0.5 µg/mL each). The automated Immunospot S6 core reader and software (CTL Europe GmbH) were used to count sfu using SmartCount™ and Autogate™ functions. Spot forming units (sfu) of stimulated cells were detected according to the manufacturer's recommendations. Specific responses were calculated after averaging duplicate wells and subtracting non-specific responses (solvent without peptides).

Reference:

1. Abravanel F, Miedouge M, Chapuy-Regaud S, Mansuy JM, Izopet J. Clinical performance of a rapid test compared to a microplate test to detect total anti SARS-CoV-2 antibodies directed to the spike protein. J Clin Virol 2020;130:104528.
